# Mind4Health: decolonizing gatekeeper trainings using a culturally relevant text message intervention

**DOI:** 10.3389/fpubh.2024.1397640

**Published:** 2024-09-02

**Authors:** Colbie Caughlan, Amanda Kakuska, Jane Manthei, Lisa Galvin, Aurora Martinez, Allyson Kelley, Stephanie Craig Rushing

**Affiliations:** ^1^Northwest Portland Area Indian Health Board, Portland, OR, United States; ^2^Allyson Kelley & Associates PLLC, Sisters, OR, United States

**Keywords:** American Indian, suicide prevention, gatekeeper training, health education, text message intervention

## Abstract

**Background:**

When a person dies by suicide, it takes a reverberating emotional, physical, and economic toll on families and communities. The widespread use of social media among youth and adolescents, disclosures of emotional distress, suicidal ideation, intent to self-harm, and other mental health crises posted on these platforms have increased. One solution to address the need for responsive suicide prevention and mental health services is to implement a culturally-tailored gatekeeper training. The Northwest Portland Area Indian Health Board (NPAIHB) developed Mind4Health, an online gatekeeper training (90 min) and text message intervention for caring adults of American Indian/Alaska Native (AI/AN) youth.

**Methods:**

The Mind4Health intervention was a multi-phase, single-arm, pre-and post-test study of users enrolled in the intervention that is available via text message (SMS) or via a 90 min online, self-paced training. We produced four datasets in this study: Mobile Commons, pre-survey data, post-survey data, and Healthy Native Youth website’s Google Analytics. The analysis included data cleaning, basic frequency counts, percentages, and descriptive statistics. Qualitative data were analyzed using thematic content analysis methods and hand-coding techniques with two independent coders.

**Results:**

From 2022 to 2024, 280 people enrolled in the Mind4Health SMS training, and 250 completed the 8-week intervention. Many messages in the sequence were multi-part text messages and over 21,500 messages were sent out during the timeframe. Of the 280 subscribers, 52 participated in the pre-survey. Pre-survey data show that 94% of participants were female, and nearly one-fourth lived in Washington state, 92% of participants in the pre-survey were very to moderately comfortable talking with youth about mental health (*n* = 48). Most participants interact with youth in grades K–12. Post-survey data demonstrate changes in knowledge, beliefs, comfort talking about mental health, and self-efficacy among participants. Mind4Health improved participant’s skills to have mental health conversations with youth and refer youth to resources in their community.

## 1 Introduction

When a person dies by suicide, it takes a reverberating emotional, physical, and economic toll on families and communities. In 2019, suicide was the fourth leading cause of death globally for people aged 15–29 years (1). In the United States, there were 48,183 suicide deaths in 2021, and suicide was the second leading cause of death among people aged 10–14 years and 20–34 years (2). Some populations in the United States have higher rates, including American Indian or Alaska Native (AI/AN) people, veterans, individuals living in rural areas, and sexual and gender minorities (2). Unresolved trauma, grief, and loss are key factors associated with suicidal ideation and attempts (2).

From 2011 to 2021, suicide death rates significantly increased among people of color. AI/AN populations had the highest increase, from 16.5 to 28.1 per 100,000, followed by Black populations, which had a 58% increase from 5.5 to 8.7 per 100,000 during this period (3). Furthermore, the COVID-19 pandemic disrupted daily life with widespread lockdowns and school closures. Meherali et al. conducted a rapid systematic review of youth and adolescent mental health during the COVID-19 pandemic, and the results showed an increase in emotional stress, fear, anxiety, and depression (4). Bridge et al. identified 5,568 American youth aged 5–24 who died from suicide in 2020 during the first year of the COVID-19 pandemic, and an estimated 3.1% (170) were AI/AN youth (5). The pandemic had negative social, economic, and cultural consequences for AI/AN communities that may have contributed to an increase in suicides during this period (5, 6).

Internet and social media platform use increased significantly during the first year of the pandemic as many utilized these tools to stay connected with peers, family, and friends during social distancing measures (7). Social media platforms, such as X, formerly known as Twitter, Facebook, Instagram, and TikTok, have become a more integral part of daily life in recent years (8). An estimated 95% of youth have access to a smartphone device; over 77% of youth used YouTube, 58% used TikTok, and 50% used Instagram applications daily according to a Pew Research Center survey conducted in 2022 (9).

With the widespread use of social media among youth and adolescents, disclosures of emotional distress, suicidal ideation, intent to self-harm, and other mental health crises posted on these platforms have increased (8, 10, 11). Gritton et al. conducted a focus group study with AI/AN youth in Washington and Oregon to examine views, interpretations, and actions taken when a peer publishes a concerning post online (10). A total of 32 AI/AN youth participated in three focus groups at community events. Three themes emerged from their findings: (1) youth often respond alone to concerning posts; (2) barriers to action, including difficulty understanding the true meaning of a concerning post and experiencing responder fatigue; and (3) the importance of confiding in a trusted adult or third-party responder when a concerning post is seen (10). Their findings identified a need for resources to help caring adults and peers intervene promptly to provide support and assistance to the person in distress (11).

To better prepare caring adults for that role, raining community gatekeepers is an evidence-based prevention strategy used to “identify and assist” people at-risk of suicide (12). Gatekeepers are community members: teachers, student peers, coaches, clergy, first responders, and other non-specialists who are trained to identify others experiencing suicidal ideation (13, 14). Community gatekeeper training programs are associated with increased knowledge to identify individuals at-risk for suicide and increased skills to intervene (13). The Question, Persuade, and Refer (QPR) gatekeeper training is the among the most widely utilized, validated trainings, endorsed by the Suicide Prevention Resource Center (15). The model teaches trainees to recognize suicidal warning signs, reduce immediate suicide risks, and connect at-risk individuals to appropriate mental health services (13). McKay et al. studied an online suicide prevention gatekeeper program focused on empowering parents to engage and support a child who exhibits suicidal ideation or suicidal behavior (16). The study took place in Victoria, Australia, with a total of 127 participants who identified themselves as parents of young people aged 12–25 years (16). The findings suggested suicide prevention training increased parents’ self-efficacy to intervene and help a suicidal individual as well as reduce the stigma around suicide.

While evaluations of gatekeeper training models are associated with improved knowledge and skills to recognize and prevent suicides, they are not always culturally-tailored to AI/AN communities, who demonstrate the highest need for such interventions (17). There is a need for decolonized gatekeeper trainings grounded in traditional knowledge and Indigenous Ways of Knowing where respect, relationship, and reciprocity serve as a guide (18). A scarcity of decolonized suicide prevention services and/or resources makes it challenging to respond and identify suicide risks and resiliency factors in AI/AN communities. Moreover, a lack of culturally tailored decolonized gatekeeper suicide intervention trainings can create barriers by using strategies that do not resonate with AI/AN culture, language, and beliefs on suicide (18–20).

Gatekeeper training programs like Question Persuade Refer (QPR), Applied Suicide Intervention Skills Training (ASIST), Native Motivational Interviewing, and Youth Mental Health First Aid for Tribal Communities and Indigenous Peoples have cultural adaptations or in some cases, are taught by AI/AN trainers (21). However, to our knowledge, the cultural relevance of these trainings have not been systematically reviewed with AI/AN stakeholders. Anecdotal data from past participants indicates that the training materials were adapted by adding AI/AN suicide rates (a surface-level adaptation) (22). However, no deeper-level inclusion of culturally-relevant teaching methods, values, or learning styles were woven into the training. These are important features of culturally responsive teaching strategies (20).

Designing with intention a culturally tailored, decolonized AI/AN-specific suicide prevention framework can encourage generational healing, kinship, and reciprocity that builds resilience, lessens vulnerability, connects to kinship systems that endure over time. Characteristics of a culturally tailored gatekeeper training for AI/AN populations would include the following: deep cultural tailoring which is developed and reviewed by AI/AN community members and experts to include cultural values, beliefs, along with surface cultural tailoring of peripheral, evidential, and linguistic components. This type of training increases cultural awareness and cultural resiliency, addresses AI/AN strengths and community risk factors, and values the lived experiences of AI/AN community members (20). This paper fills an essential gap in the literature by documenting the development and preliminary outcomes of a decolonized, AI/AN stakeholder-reviewed, culturally-centered gatekeeper training for caring adults working with AI/AN youth, delivered online and via Short Message Service (SMS) text messaging.

### 1.1 Designing culturally relevant gatekeeper trainings

The Northwest Portland Area Indian Health Board (NPAIHB) is a regional, tribal nonprofit organization serving the 43 federally recognized Tribes in Washington, Oregon, and Idaho. The Northwest Tribal Epidemiology Center is housed under NPAIHB and provides support through research, surveillance, and public health capacity building in partnership with the Northwest Tribes. The gatekeeper trainings discussed in this paper were designed by the THRIVE suicide prevention project, Behavioral Health, and the Adolescent Health teams at the NPAIHB. NPAIHB recruited participants, delivered the SMS gatekeeper text messages, and led the design of data collection tools, data collection, and data analysis.

In 2017, the NPAIHB partnered with the Social Media & Adolescent Health Research Team to design, implement, and evaluate the first iteration of this gatekeeper training: Responding to Concerning Posts on Social Media (22, 23). The evaluation of the web-based training included 70 adults. Adults in Arm 1 watched a 30-min gatekeeper video and read handouts. Adults in Arm 2 watched the 30-min training video, reviewed accompanying training handouts, and participated in an interactive role-play scenario with a coach that took place via text message. Evaluation findings demonstrate that the intervention produced significant improvements across several areas, including confidence in starting conversations, confidence in containing a person who posted something concerning, and confidence in recommending support services to youth (22). This was the first gatekeeper training for adults that provided guidance for responding to concerning posts on social media with AI/AN youth (22).

During the COVID-19 pandemic, the NPAIHB updated the Responding to Concerning Posts gatekeeper training and made several improvements based on user feedback from the pilot study. The primary updates consisted of adding additional opportunities for participant engagement, extending the duration of the training, and developing animations that role-model gatekeeping skills and culturally tailored mental health strategies. For example, participants wanted more practical tips on how to respond to a youth who was contemplating suicide.

### 1.2 The Mind4Health intervention

The new iteration was re-named Mind4Health. The decolonized Mind4Health online gatekeeper training intervention focuses on three behavior change areas: respond, heal, and grow, and utilizes aspects of Bandura’s self-efficacy theory and the theory of planned behavior (24). Traditional suicide gatekeeper interventions focus on changing knowledge, beliefs, stigma, and self-efficacy (25). Our decolonized theoretical framework focuses on the same learning outcomes, but also recognizes the impacts of colonization, the importance of lived experiences and stories, and supports a strengths-based approach to intervention and healing. Since its launch in July 2022, over 280 caring adults have signed up for the new Mind4Heath text message campaign.

**Figure 1:**
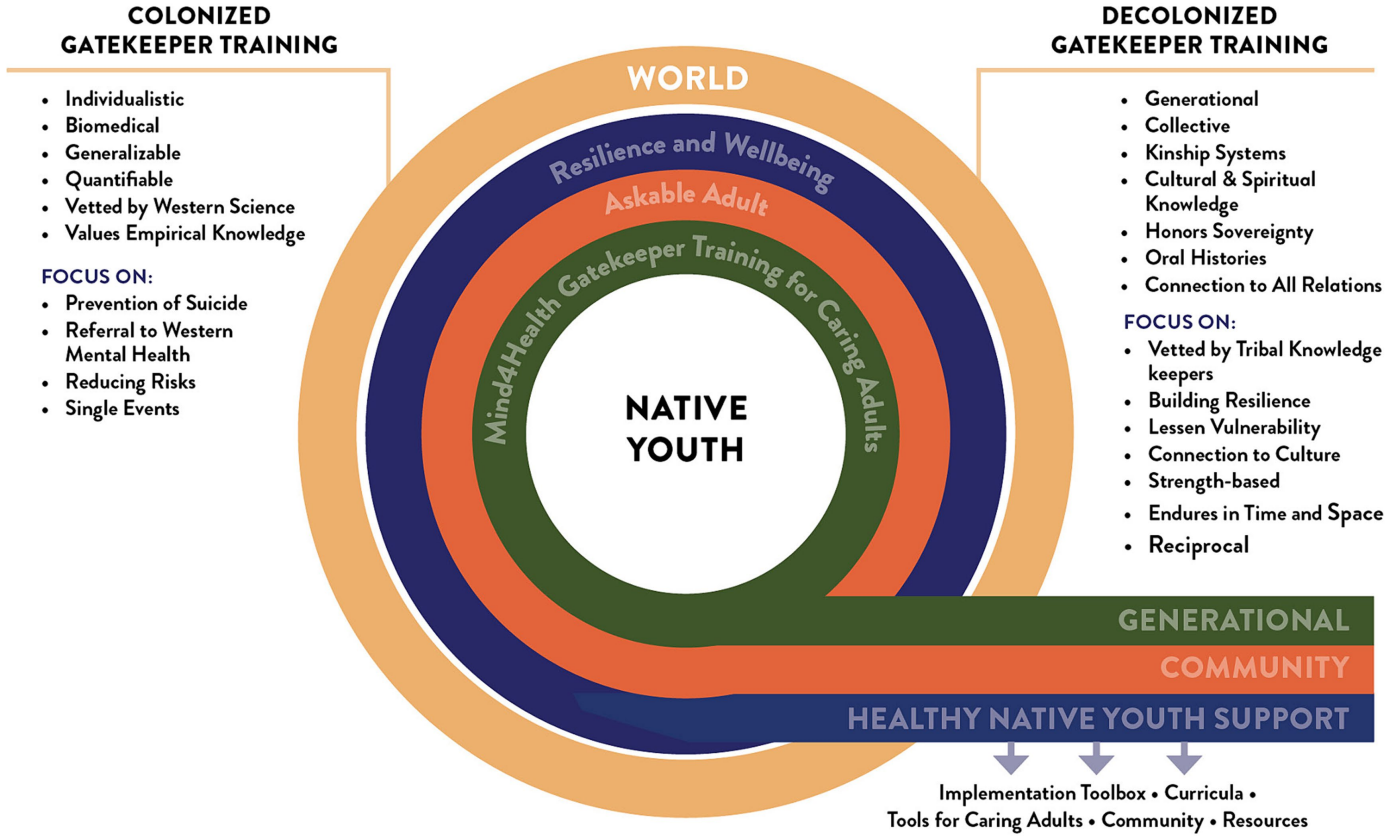
Mind4Health decolonized gatekeeper model.

During the update, developers added three new role model videos to the training, demonstrating the skills needed to be an “Askable Adult,” incorporated a text message sequence to practice and build up mental health skills over time, and incorporated Indigenous Ways of Knowing and Healing to support youth’s mental health. To our knowledge, this is the only suicide prevention gatekeeper training offered via SMS (see Figure 1).

## 2 Materials and methods

### 2.1 Mind4Health components

The Mind4Health intervention includes two components. The first intervention component is a SMS text message sequence. The second intervention component is an online self-paced gatekeeper training, supplementing the SMS series.

Frequently asked questions about Mind4Health:

- Who can enroll in Mind4Health? Mobile Commons, the software used to deliver the text messages, is most reliable in the U.S.—in the lower 48 States. However, the online training components are available worldwide.
- Who should enroll? Mind4Health is designed for parents, coaches, counselors, and families who work with Native youth.
- What is the cost? There is no cost to participants.
- How is Mind4Health delivered? Online and/or via text message.
- What happens at enrollment? Caring adults receive 1–2 text messages per week for 8-weeks with role-model videos, conversation starters, tips, and words of encouragement that are culturally tailored with an Indigenous lens (22).
- What happens upon completion? Upon completion of the text messages, participants are asked to complete an online post-survey and receive a $30 gift card.
- What topics does Mind4Health cover? Topics cover gatekeeping and referral skills and focus on the themes of Response, Heal, and Grow.
- How is Mind4Health evaluated? Trainees complete a pre- and post-survey. Google Analytics and Mobile Commons data document dissemination and reach.

### 2.2 Study design

The Mind4Health intervention was a multi-phase, single-arm, pre-and post-test study of users enrolled in the gatekeeper intervention available via SMS and a 90-min online, self-paced training. All data collection tools were reviewed by the Portland Area Indian Health Service Institutional Review Board (PA IHS IRB) in Portland, OR (*PI:* Craig Rushing) and the evaluation findings were approved for publication. The pre & post-survey tools collected basic demographic information required by the funder (SAMSHA) when conducting gatekeeper trainings—including tribal affiliation, state, gender, primary professional role. The post-survey also included questions adapted from our prior evaluation of the Concerning Post training, which Mind4Health is modeled after (18).

#### 2.2.1 Participant recruitment and eligibility

Adults were recruited via the NPAIHB’s social media channels (Facebook, text message, Instagram), THRIVE’s listserv reaching Tribes, tribal health organizations, Indian education and human service organizations that serve AI/AN teens and young adults. Adults were asked to text the keyword “Mind4Health” to the shortcode 65664. This prompted a series of eligibility and consent text messages, including a link to an online consent form for more information about Mind4Health.

The study included self-identified adults. All participants were required to have a cell phone with text message capabilities. Those who enrolled were asked to complete the pre-survey online (SurveyMonkey). Participants could opt out of the SMS sequence or online training at any time.

#### 2.2.2 Delivery of the online training and text messaging intervention

Messages were delivered in a pre-scheduled format, which was based on their enrollment date. The first message in the sequence arrived 3 days after signing up. We tracked message delivery by the participant’s phone number, delivery date, and content. Some messages also included link tracking, which recorded engagement through unique link clicks, showing how many times a given URL resource was opened.

### 2.3 Datasets

We produced four datasets in this study. The first dataset came from Mobile Commons and documents the enrollment and reach of the intervention. The second dataset represents pre-survey data; the third dataset includes post-survey data. The pre-survey includes demographic questions, the age of the youth they work with, their comfort level in talking about mental health, and their role in the community. The post-survey includes questions about how prepared they feel to be an askable adult after receiving the intervention, comfort talking about mental health, helpfulness of the resources provided, help-seeking and referral skills, behavior changes because of the intervention, and areas for improvement. Last, the Healthy Native Youth website’s Google Analytics dashboard provides information about the number of participants accessing the website and online training. Table 1 highlights dataset types, constructs, and survey questions (see Supplementary material for the actual surveys).

**Table 1:** Dataset type, constructs, and measure item/survey questions.

Dataset type	Construct	Survey question
Mobile commons	Reach	How many people enrolled in and completed the SMS series?
Pre-survey	Demographics	What is your Tribal affiliation?
	Demographics	What state do you live in?
	Demographics	What is your gender?
	Demographics	How old are the majority of youth you interact with?
	Mental health	How comfortable are you talking with youth about mental health?
	Demographics	What is your main role in your community?
Post-survey	Beliefs	How prepared do you feel to be an “Askable Adult”?
	Reluctance/Stigma	How comfortable are you talking with youth about mental health?
	Knowledge	How helpful were videos in modeling the steps and skills?
	Knowledge	How helpful were the links to articles and other resources?
	Self-efficacy	How likely are you to model and practice mental health self-care?
	Reluctance	Did this improve the mental health conversations you are having or help-seeking and referral skills?
	Self-efficacy	What will you do as a result of the text messages?
	Demographics	What is your main role in your community?
	Intervention improvements	If you could change or improve the series, what would it be?
Google analytics	Reach	How many times was the online Mind4Health training page and resource page visited on the Healthy Native Youth website?

#### 2.3.1 Data analysis

Data were downloaded into SPSS version 29.0. The analysis included data cleaning, basic frequency counts, percentages, and descriptive statistics. An independent-sample, non-parametric statistical test (Fisher’s Exact Test) was used to examine the differences in participants’ responses to the question: “How comfortable are you talking with youth about mental health?” (26). Qualitative data were analyzed using thematic content analysis methods and hand-coding techniques with two independent coders (27). Qualitative analysis followed published guidelines and included open coding text responses from surveys to create a codebook. Coders reviewed data systematically for themes first to explore concepts and categories in the data and then group data into themes based on their similarity (27). Quantification was used to summarize the frequency of themes identified.

## 3 Results

From 2022 to 2024, 280 people enrolled in the Mind4Health SMS training, and 250 completed the 8-week intervention. Many messages in the sequence were multi-part text messages and over 21,500 messages were sent out during the timeframe (See Supplementary material for example of SMS texting sequence). We received 980 responses, including text message reactions (i.e., thumbs-up emojis or replies with “loved this message”) and opt-outs. There was a 38% click-through rate among the links that were tracked for engagement. Mind4Health pre- and post-survey data show improvements in knowledge. Participants shared positive feedback about resources and feel more prepared to be an askable adult. The Mind4Health Gate Keeper Training and Tools for Caring Adults are some of the most viewed resources on the Healthy Native Youth website.

### 3.1 Mind4Health pre-survey

Fifty-two subscribers completed the pre-survey. Pre-survey data show that 94% of participants were female, and nearly one-fourth lived in Washington state, 92% of participants in the pre-survey were very to moderately comfortable talking with youth about mental health (*n* = 48). Most participants interact with youth in grades K–12. Roles varied, from educators and mentors to parents and family members in Table 2.

**Table 2:** Demographics of pre-survey Mind4Health participants (*N* = 52).

Variable		Participants
Gender, *n* (%)		
Female	49 (94%)	Male	2 (4%)	Two spirit	1 (2%)
What state do you live in? *n* (%)		
Washington	12 (23%)	California	5 (10%)	Montana	5 (10%)	Oregon	5 (10%)	Arizona	4 (8%)	Oklahoma	4 (8%)	Alaska	3 (6%)	Michigan	3 (6%)	New York	2 (4%)	South Dakota	2 (4%)	Colorado	1 (2%)	Indiana	1 (2%)	Missouri	1 (2%)	Nebraska	1 (2%)	New Mexico	1 (2%)	Texas	1 (2%)	Wisconsin	1 (2%)
Age of the youth you interact with? *n* (%)		
Elementary school	15 (29%)	Middle school	14 (27%)	High school	19 (37%)	Older	4 (8%)
What is your main role in the community? *n* (%)		
Other	20 (38%)	Parent/family member	11 (21%)	Behavioral health staff (including substance abuse prevention)	7 (13%)	Social worker, case worker, care coordinator, child welfare staff	6 (12%)	Peer support specialist, peer mentor	4 (8%)	Culture keeper or traditional healer	2 (4%)	Medical provider (including dental)	1 (2%)	Substance abuse counselor	1 (2%)

“Other” included educators, mentors, school counselors, victim advocates, and community organizers/navigators.

### 3.2 Mind4Health post survey

Participant roles in the community at post-survey were similar to the pre-survey, with parents, family members, educators, and outreach roles being most frequent in Table 3. Ninety subscribers completed the post-survey.

**Table 3:** Roles of community members at post-test (*N* = 90).

Variable		Participants
What is your main role in the community? *n* (%)		
Parent/family member	27 (30%)	Other	25 (28%)	Social worker, case worker, care coordinator, child welfare staff	14 (16%)	Behavioral health staff (including substance abuse prevention)	12 (13%)	Tribal council member/tribal elder	4 (4%)	Peer support specialist, peer mentor	4 (4%)	Culture keeper or traditional healer	4 (4%)

“Other” included educators, mentors, program directors and managers, violence prevention advocates, and community outreach navigators.

**Figure 2:**
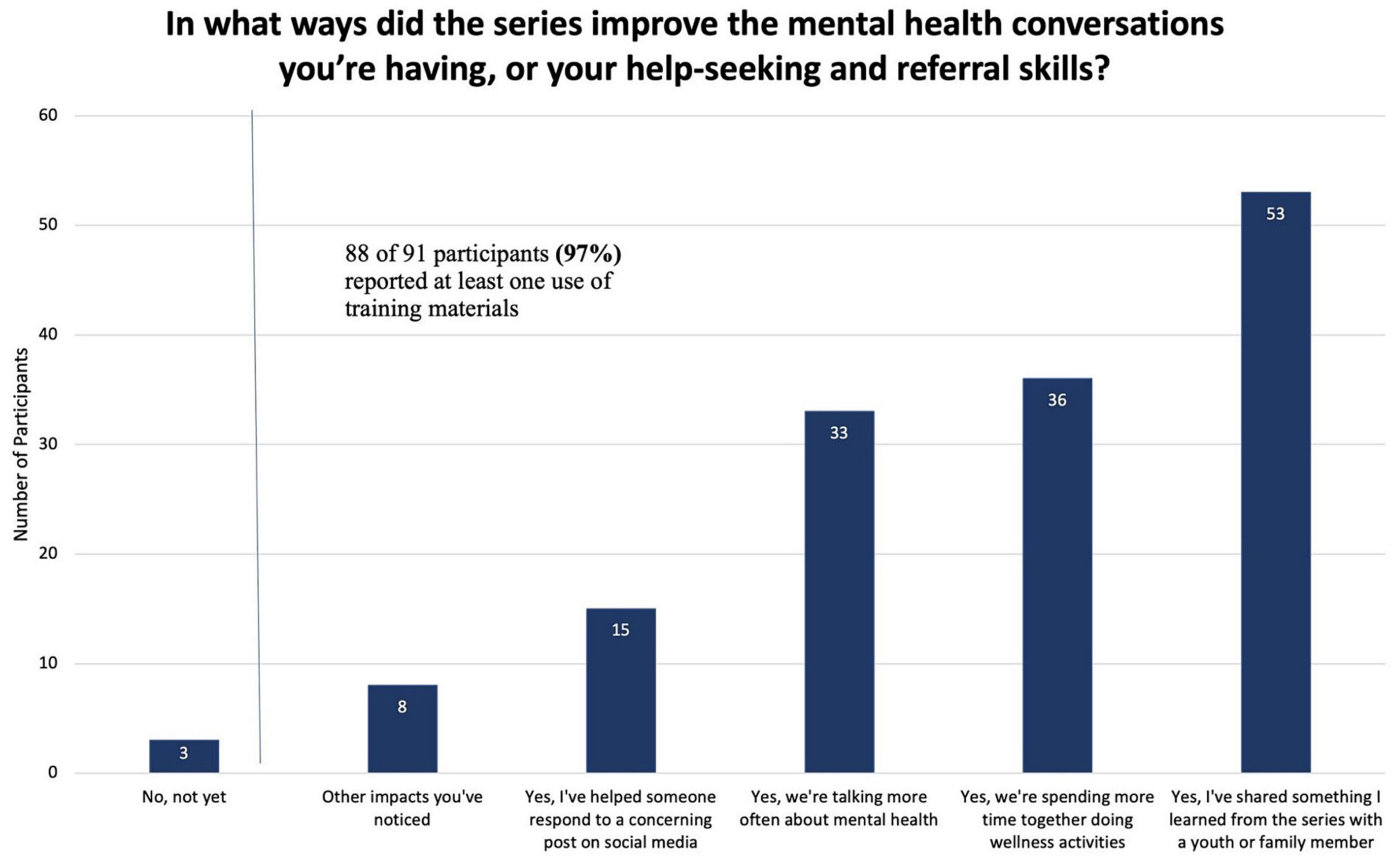
Improvements in mental health conversations.

Post-survey data demonstrate changes in knowledge, beliefs, comfort talking about mental health, and self-efficacy among participants. Mind4Health improved participant skills to have mental health conversations with youth and refer youth to resources in their community (see Figure 2). However, the comfort level of participants talking about mental health did not change significantly from pre- to post-survey in Table 4.

**Table 4:** Comfort talking about mental health across pre- (*N* = 52) and post-test (*N* = 90).

Variable		Pre-test	Post-test	*p* value
How comfortable are you talking with youth about mental health? *n* (%)				
Very	29 (56%)	52 (58%)	0.9772	Moderately	19 (37%)	30 (33%)	A little	4 (8%)	8 (9%)	Not at all	0 (0%)	0 (0%)

Differences in participants’ responses to the question: “How comfortable are you talking with youth about mental health?” are highlighted in Table 4. Participants were provided with responses of “not at all,” “a little,” “moderately,” or “very.” As shown in Table 4 above, there was no statistically significant difference in the distribution of participants’ levels of comfort talking with youth about mental health pre vs. post (*p* = 0.9772).

After completing the series, 75% of participants felt more prepared to be an askable adult and 42% found the videos very helpful, and 52% found the articles and resources very helpful, noted in Table 5.

**Table 5:** Frequency of Mind4Health participant responses (*N* = 91).

**Overall, how prepared do you feel to be an “Askable Adult” after receiving the text messages?*n* (%)**	**Participants**
More prepared than I was before	75 (82%)
**If you viewed at least one of the videos, how helpful were they at modeling the steps and skills involved in having sensitive mental health conversations with youth?*n* (%)**	**Participants**
Very helpful	42 (47%)
Helpful	38 (42%)
**If you opened the links to articles and other resources, how helpful were they?*n* (%)**	**Participants**
Very helpful	47 (52%)
Helpful	39 (43%)
**After receiving the messages, how likely are you to model and practice Mental Health self-care?*n* (%)**	**Participants**
I am likely to model or practice mental health self-care	84 (92%)
**As a result of the text messages…,*n* (%)**	**Participants**
I saved both the Youth Support Resources or the Crisis Text Line to my phone	43 (47%)

See Supplementary material for a list of all responses.

In the final question, participants were asked: We appreciate your honest feedback. If you could change or improve something about the series, what would it be? Qualitative recommendations for changes to improve the Mind4Health intervention included: please continue the service, send “more frequent texts,” suggestions for additional content and resources—like “stickers to send out would be nice”—and providing clarification on the characters depicted in the role model video, “sometimes using names confused me…,” included in Table 6.

**Table 6:** Qualitative themes to improve Mind4Health.

Theme	Responses
Continue text messaging service	“More frequent texts.”
	“I really love the text reminders for mental health and quotes shared.”
	“More info text messages to keep the wellness aspect compactly on my mind to teach others and myself.”
	“Keep those texts coming forever.”
	“I love getting these texts. They seem to come at just the right time. I have shared them with peers to help them with conversations with their students as well!”
Include additional content	“If I could improve something it would be more talking point questions for families, or more wellbeing activities.”
	“More text resources. I love the encouragement texts but loved the resources more. “
	“More pics.”
	“To model their behavior.”
	“Stickers to send out would be nice.”
	“Should be shared on social media like a highlight of what is shared.”
Include additional resources	“Include additional resources.”
	“Podcast or video series.”
	“More inspirational quotes or advice.”
	“All the resources are excellent and culturally relevant. Please keep sending them!”
	“Increase face-to-face communication by having conversations about mental health. If face-to-face not possible (because of COVID) there should be FaceTime or Zoom meetings.”
Provide clarification	“Be aware of using stigmatizing language like substance “abuse” or words like “addict” can be hurtful.”
	“Sometimes using names confused me that someone else was texting me.”

### 3.3 Mind4Health dissemination

From 2022 to 2024, the Mind4Health “Tools for Caring Adults” webpage had 1,307 pageviews, from 730 users. The number of pageviews per user was 1.79, and the average engagement time on the page was 58 s. During that period, the Mind4Health “Tools for Caring Adults” resource page was the 14th most visited page on the Healthy Native Youth website: https://www.healthynativeyouth.org/resources/mind4health/.

From 2022 to 2024, the Mind4Health Gatekeeper Training page had 1,007 pageviews, from 573 users. The number of pageviews per user was 1.76, and the average engagement time on the page was 56 s. During that period, the Mind4Health Gatekeeper Training curricula page was the 18th most visited page on the Healthy Native Youth website: https://www.healthynativeyouth.org/curricula/mind4health-training/.

The Articulate Software used to design and host the 90-min online training does not track “views” or participant engagement (i.e., embedded polls or reflection activities) and are thus not included in this paper.

## 4 Discussion

Outcomes from this study show several benefits from the Mind4Health training among intervention participants. Participants felt more prepared to have mental health conversations after the training. Resources, links, and articles were helpful and utilized by participants. Notably, participants reported they shared information and resources from the Mind4Health resource page with youth and other family members. Participants increased their knowledge, confidence, and skills to recognize a person in mental health distress and are better able to link those people to appropriate resources. Findings from this evaluation are unique; no other studies cite the use of a decolonized gatekeeper training to address suicide in AI/AN populations. The results of this study support the continued use of decolonized gatekeeper trainings to address the suicide epidemic by building community-based knowledge, addressing stigma that often surrounds mental health help-seeking, and growing the self-efficacy of trusted, askable adults.

Recommended improvements to the Mind4Health intervention were minimal and are likely because the curriculum was designed and vetted by AI/AN stakeholders and updated from previous work with AI/AN communities. Some participants wanted to receive additional text messages, others felt additional content, resources, and information would be helpful. These recommendations will be integrated into future iterations of the Mind4Health training.

Our study findings are supported by other evaluations of gatekeeper trainings to prevent suicide (28, 29). Such studies demonstrate the importance of gatekeeper training for building knowledge, and self-efficacy, addressing stigma, and providing resources to lessen suicide risk. However, these evaluations were not conducted using a decolonized gatekeeper approach. Findings from colonized trainings are limited because they focus narrowly on quantitative outcomes and effect sizes, and value Western educational systems and degrees as a benchmark for assessing knowledge and self-efficacy. This is in contrast to a decolonized model, where a generational connection to all relations, lived experience, subjective data and cultural resilience are the focus.

Every AI/AN community has a unique history, culture, language, and tradition. A one-size-fits-all approach to gatekeeper training interventions is not possible. In fact, standardized approaches to developing and implementing gatekeeper training with AI/AN populations may not be effective if they are incongruent with the experiences of AI/AN youth and families in those communities. Most do not recognize the etiology of suicide and distress leading to suicide, including colonization, residential boarding schools, assimilation, structural violence, and oppression (18). Biomedical and individualistic approaches to addressing suicide in AI/AN populations are limited in their effectiveness and sustainability because they fail to honor the significance of relational and spiritual connections, world view, historical and intergenerational trauma, and kinship systems that are at play in communities, not always measured or counted in the Western context.

Stigma and reluctance to discuss suicide are common, especially in AI/AN communities (29). Culturally-tailored SMS and online gatekeeper interventions such as Mind4Health may be more effective in reaching people because user-engagement is anonymous and can be completed at the participant’s convenience and staggered readiness levels; they do not require live or in-person conversations and roleplays with others. Changing the focus and language from suicide to vulnerability may be necessary to fully decolonize gatekeeper trainings. In Mind4Health, the focus is on identifying and building local support networks and empowering people to tap into local and national resources before a crisis occurs. To help build help-seeking skills among those at-risk, the NPAIHB also designed and delivers Caring Text Messages (CTM), an evidence-based intervention modeled after Caring Contacts, which has been shown to prevent suicide in a variety of settings. The Caring Text Messages were co-developed with AI/AN teens, college students, and veterans, and were designed to increase protective factors, like cultural pride, hope, and self-esteem. While doing so, the CTMs offer participants a personal connection to peers they could relate to (30).

The Promoting Community Conversations About Research to End Suicide approach is another example of a decolonized intervention to address a vulnerability that focuses on the role of stories, connections, hope, culture, and belonging (31). By following a broader path of prevention and response, decolonized interventions have the potential to reach entire communities and address not just suicidality but the broader social and structural determinants of health that lead to deaths of despair in AI/AN communities (32).

### 4.1 Strengths and limitations

While this study has multiple strengths, a few limitations should be noted based on the preliminary data collected. First, participants assessed at the pre-test were unable to be paired at the post-test due to the lack of a reliable and unique identifying variable. Although we employed Fishers’ Exact Test to examine differences (25), this does not tell the entire story. Most participants enrolling in the Mind4Health series have interests, knowledge, and experiences with mental health conversations. Participants were moderate to very comfortable before and after the intervention, and this may be related to how individuals were recruited for the series and their involvement with THRIVE and other mental health promotion programs. Moreover, participants could select to complete the online self-paced webinar and the SMS sequence, alone or combined. This study does not differentiate between those who completed both versus one. Data are based on self-report measures, and social desirability bias or recall bias may be present.

There was a high level of competence of participants at the pre-survey to have mental health conversations with youth. This led to no significant change in pre- and post-survey data related to comfort level with having mental health conversations. Qualitative data reported by trainees does not represent all possible areas for improvement and does not include responses of appreciation or no comment. These findings may not be generalizable to all Tribal communities, trainees, and professionals because of the small sample size, purposive recruitment methods, and limited digital access and inequality that is pervasive in AI/AN communities. Even with these limitations in mind, this preliminary report demonstrates promising outcomes for the first decolonized gatekeeper training to prevent suicide in AI/AN communities, delivered online or via text message.

## 5 Conclusion

The findings from this study suggest promising results from the first-ever gatekeeper training for AI/AN caring adults, delivered via text message and a 90-min online training. As policymakers, professionals, and leaders consider how to address the suicide epidemic in this population, they must also address the broader social conditions that increase the risk of suicide. Strengthening the knowledge and skills of “Askable Adults” in AI/AN communities is the first step in addressing the epidemic. Future efforts are necessary to continue these conversations while addressing beliefs about mental health and the reluctance many feel to ask another person if they are considering suicide. Building the self-efficacy of gatekeepers to ask these difficult questions, while directing youth and families to culturally centered resources like those found on the Healthy Native Youth website, is the first step in a decolonized prevention approach.

## Data Availability

The original contributions presented in the study are included in the article/Supplementary material, further inquiries can be directed to the corresponding author.

## Supplementary material

Supplementary Material 1 Pre-survey.

Supplementary Material 2 Post survey.

Supplementary Material 3 Selected messages from the Mind4Health SMS texting sequence.

Supplementary Material 4 Full list of responses from Table 5. Frequency of Mind4Health participant responses (*N* = 91).
