# Supplementary material for: Mind4Health: decolonizing gatekeeper trainings using a culturally relevant text message intervention
Source: Front Public Health. 2024 Sep 2;12:1397640. doi: 10.3389/fpubh.2024.1397640 (PMC11403716; doi:10.3389/fpubh.2024.1397640)
Supplement: Supplementary Material 1 — Pre-survey. [file Data_Sheet_1.PDF]

Supplementary File 1. Pre-Survey

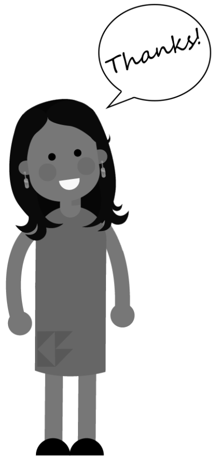

Mind4Health Pre-Survey

Consent

Goal. We are interested in learning whether our text message service supports parents, educators, and caring adults.

Consent. You are being asked to participate in a text message survey that is being conducted by the Northwest Portland Area Indian Health Board (PI: Stephanie Craig Rushing, PhD, MPH). The survey will take less than 10 minutes to complete.

There are no right or wrong answers to the questions; your honest feedback will help us improve our programs for future users.

Risks. There is a small risk that your information could be disclosed. This is unlikely given the steps that we will take to protect your privacy and keep all information confidential.

Benefits. The information we collect may not benefit you directly, but what we learn from your feedback will be used to improve the quality of our programs and services.

The survey is confidential. Your text message survey answers will be linked to your cell phone number. All of the data will be aggregated so that no one person or tribe can be identified.

Your participation is voluntary. You may choose not to participate in the survey, you may stop responding at any time, or you may skip any questions that you do not want to answer. Your completion of the survey serves as your voluntary agreement to participate in this research. Questions about the purpose of the research can be directed to me, at scraig@npaihb.org.

This survey has been reviewed by the Portland Area (PA) Indian Health Services' (IHS) Institutional Review Board (IRB), a tribal committee that is responsible for protecting the rights and welfare of research participants and tribal members. If you have any concerns about your rights as a participant, please contact Thomas Weiser, MD, MPH at 1-877-664-0644. Dr. Weiser is the Co-Chair of the PA IHS IRB, which has reviewed this project.

Thank you for your time and participation!

1. What is your Tribal Affiliation?

2. What State do you live in?

3. What is your gender?

- Female
- Male
- Transgender
- Other (please specify):

4. How old are the majority of youth you interact with?

- Elementary school
- Middle school
- High school
- Older

5. How comfortable are you talking with youth about mental health?

- Very
- Moderately
- A little
- Not at all

6. What is your **main role** in your community? Please select the one role that fits best:

- tribal council member/tribal elder
- peer support specialist/peer mentor
- behavioral health staff (including substance abuse/suicide prevention)
- culture keeper or traditional healer
- parent/family member
- medical provider (including dental)
- substance abuse counselor
- social worker/caseworker/care coordinator/child welfare staff
- law enforcement/first responder
- jail/prison/detention center staff
- clergy
- Other (please specify)

7. Do you have any comments, questions, or concerns before we begin?
