# Supplementary material for: Mind4Health: decolonizing gatekeeper trainings using a culturally relevant text message intervention
Source: Front Public Health. 2024 Sep 2;12:1397640. doi: 10.3389/fpubh.2024.1397640 (PMC11403716; doi:10.3389/fpubh.2024.1397640)
Supplement: Supplementary Material 2 — Post survey. [file Data_Sheet_2.PDF]

Supplementary File 2. Post Survey

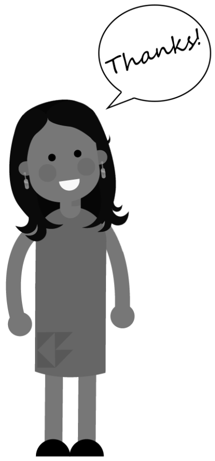

Mind4Health Post Survey

1. Overall, how prepared do you feel to be an "Askable Adult" (starting the conversation and creating safe spaces to talk to youth about mental health) after receiving the text messages?

- ◯ Less prepared than I was before
- ◯ As prepared as I was before
- ◯ More prepared than I was before

2. How comfortable are you talking with youth about mental health?

- ◯ Very
- ◯ Moderately
- ◯ A little
- ◯ Not at all

3. If you viewed at least one of the videos, how helpful were they at modeling the steps and skills involved in having sensitive MH conversations with youth?

- ◯ Very helpful
- ◯ Helpful
- ◯ Not helpful
- ◯ N/A: I did not watch the videos

4. If you opened the links to articles and other resources, how helpful were they?

- ◯ Very helpful
- ◯ Helpful
- ◯ Not at all helpful
- ◯ N/A: I did not open the linked articles or resources

5. After receiving the messages, how likely are you to model and practice Mental Health self-care?

- ◯ I am **not** likely to model or practice MH self-care
- ◯ I am **not sure** whether I will model or practice MH self-care
- ◯ I am **likely** to model or practice MH self-care

6. Did the series improve the mental health conversations you're having or your help-seeking and referral skills? (select all that apply)

- ☐ No, not yet
- ☐ Yes, we're talking more often about mental health
- ☐ Yes, we're spending more time together doing wellness activities
- ☐ Yes, I've shared something I learned from the series with a youth or family member
- ☐ Yes, I've helped someone respond to a concerning post on social media
- ☐ Other impacts you've noticed (please specify)

7. As a result of the text messages:

- ◯ I **did not** save the Youth Support Resources or the Crisis Text Line to my phone
- ◯ I **saved** the Youth Support Resources to my phone
- ◯ I **saved** the the Crisis Text Line to my phone
- ◯ I **saved both** the Youth Support Resources or the Crisis Text Line to my phone

8. What is your **main role** in your community? Please select the one role that fits best:

- ◯ tribal council member/tribal elder
- ◯ peer support specialist/peer mentor
- ◯ behavioral health staff (including substance abuse/suicide prevention)
- ◯ culture keeper or traditional healer
- ◯ parent/family member
- ◯ medical provider (including dental)
- ◯ substance abuse counselor
- ◯ social worker/caseworker/care coordinator/child welfare staff
- ◯ law enforcement/first responder
- ◯ jail/prison/detention center staff
- ◯ clergy
- ◯ Other role (please specify)

9. We appreciate your honest feedback. If you could change or improve something about the series - what would it be?

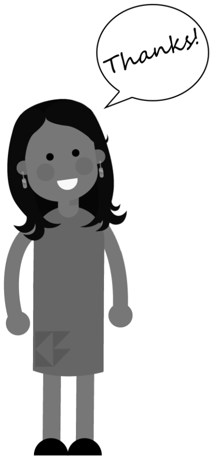

Mind4Health Post Survey

10. Thank you for sharing your feedback with us! If you would like to receive a $30 Amazon gift card, please provide your **email**.
