# Supplementary material for: Mind4Health: decolonizing gatekeeper trainings using a culturally relevant text message intervention
Source: Front Public Health. 2024 Sep 2;12:1397640. doi: 10.3389/fpubh.2024.1397640 (PMC11403716; doi:10.3389/fpubh.2024.1397640)
Supplement: Supplementary Material 3 — Selected messages from the Mind4Health SMS texting sequence. [file Data_Sheet_3.docx]

| **Selected Messages from the Mind4Health SMS Sequence** | | |
| --- | --- | --- |
| **Message Number** | **Message Body** | **Attachment** |
| 3 | Take a moment to fill your cup. Before we can help others, we must make a habit of taking care of ourselves. Take this moment to light some sage and call on the strength of your ancestors. | 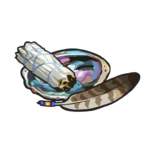 |
| 13 | The second step to starting the convo is to listen and ask questions. Validate youth's feelings or their concern about a friend. And, let them know what you plan to do next. Watch Tommy help a youth with a concerning social media post. https://www.youtube.com/watch?v=eRodoYQiIwc | 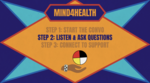 |
| 18 | Now is a great time to add the Crisis Text Line to your phone (Text: NATIVE to 741 741). You never know when you might need it to share with others.  And, save a copy to your phone of our Youth Resources list that includes a Safety Plan. Here's the link: https://linktr.ee/npaihbthrive?utm_source=qr_code |  |
| 31 | Trauma and fear create detachment and pain, that can be passed down from generation to generation. The antidote is connection; Connection to your Sacred Fire, Mother Earth, Father Sky, your community and culture. |  |

Supplementary File 3. Selected Messages from the Mind4Health SMS Texting Sequence
