# Supplementary material for: Mind4Health: decolonizing gatekeeper trainings using a culturally relevant text message intervention
Source: Front Public Health. 2024 Sep 2;12:1397640. doi: 10.3389/fpubh.2024.1397640 (PMC11403716; doi:10.3389/fpubh.2024.1397640)
Supplement: Supplementary Material 4 — Full list of responses from Table 5. Frequency of Mind4Health participant responses (N = 91). [file Data_Sheet_4.docx]

**Supplementary Table 1. Full List of Responses from Table 5. Frequency of Mind4Health Participant Responses (N = 91)**

| **Overall, how prepared do you feel to be an “Askable Adult” after receiving the text messages?, n (%)** | | | | **Participants** | |  |  |  |
| --- | --- | --- | --- | --- | --- | --- | --- | --- |
|  | More prepared than I was before | | | 75 (82%) | |  |  |  |
|  | As prepared as I was before | | | 14 (15%) | |  |  |  |
|  | Less prepared than I was before | | | 2 (2%) | |  |  |  |
| **If you viewed at least one of the videos, how helpful were they at modeling the steps and skills involved in having sensitive mental health conversations with youth?, n (%)** | | | | **Participants** | |  |  |  |
|  | | Very helpful | | 42 (47%) | |  |  |  |
|  | | Helpful | | 38 (42%) | |  |  |  |
|  | | Not helpful | | 0 (0%) | |  |  |  |
|  | | N/A: I did not watch the videos | | 10 (11%) | |  |  |  |
| **If you opened the links to articles and other resources, how helpful were they?, n (%)** | | | **Participants** | | |  |  |  |
|  | Very helpful | | 47 (52%) | | |  |  |  |
|  | Helpful | | 39 (43%) | | |  |  |  |
|  | Not helpful | | 0 (0%) | | |  |  |  |
|  | N/A: I did not open the linked articles or resources | | 5 (5%) | | |  |  |  |
| **After receiving the messages, how likely are you to model and practice Mental Health self-care?, n (%)** | | | | | **Participants** | | |  |
|  | I am likely to model or practice mental health self-care | | | | 84 (92%) | | |  |
|  | I am not sure whether I will model or practice mental health self-care | | | | 7 (8%) | | |  |
|  | I am not likely to model or practice mental health self-care | | | | 0 (0%) | | |  |
| **Self- efficacy. As a result of the text messages…, n (%)** | | | **Participants** | | |  |  | |
|  | I saved both the Youth Support Resources or the Crisis Text Line to my phone | | 43 (47%) | | |  |  | |
|  | I saved the Youth Support Resources to my phone | | 17 (19%) | | |  |  | |
|  | I saved the Crisis Text Line to my phone | | 13 (14%) | | |  |  | |
|  | I did not save the Youth Support Resources or the Crisis Text Line to my phone | | 18 (20%) | | |  |  | |
